# Continental Assessment of Work-Related Musculoskeletal Disorders Prevalence Among Surgeons: Systematic Review and Meta-Analysis

**DOI:** 10.3390/jfmk10020221

**Published:** 2025-06-09

**Authors:** Philippe Gorce, Julien Jacquier-Bret

**Affiliations:** 1 Université de Toulon, CS60584, CEDEX 9, 83041 Toulon, France; gorce@univ-tln.fr; 2 International Institute of Biomechanics and Occupational Ergonomics, Avenue du Docteur Marcel Armanet, CS 10121, 83418 Hyères Cedex, France

**Keywords:** musculoskeletal disorders, prevalence, body area, healthcare professionals, quality of life, meta-analysis, systematic review, America, Asia, Europe

## Abstract

**Background**: Work-related musculoskeletal disorders (WMSDs) are very prevalent among surgeons worldwide. The aim was to investigate the overall and body area WMSD prevalence (proportion of surgeons suffering from WMSD during their practice) by continent throughout a systematic review and meta-analysis. **Methods**: Three open databases were scanned without a date limit until 31 December 2024 to select relevant studies. The results were reported according to the Preferred Reporting Items for Systematic Reviews and Meta-Analyses guidelines. **Results**: Among the 20,486 items, 68 articles with a total of 17,188 surgeons were included, distributed as follows: 36 studies in America, 15 in Asia, 16 in Europe, and 1 in Oceania. Overall prevalence was 77.6% in Asia (95% CI: 67.3–87.9%), 73.1% in Europe (95% CI: 60.3–86.0%), and 62.8% in America (95% CI: 57.0−68.6%). The most exposed areas were the neck, upper and lower back, and shoulder, with prevalence ranging from 30 to 50%. The ranking differed according to continent. In America, neck/shoulder WMSD and overall prevalence were negatively correlated to years of experience (r^2^ = 0.182 and r^2^ = 0.240, *p* < 0.05) and to the number of cases treated per week prevalence (r^2^ = 0.794, *p* < 0.05), respectively. A positive correlation was observed between the elbow WMSD prevalence and age (r^2^ = 0.365, *p* < 0.05). In Europe, a negative correlation was highlighted between the overall WMSD prevalence and age (r^2^ = 0.599, *p* < 0.05). Another positive correlation was identified between shoulder WMSD prevalence and years of experience (r^2^ = 0.735, *p* < 0.05). **Conclusions**: To improve surgeons’ quality of work life, further research is needed to develop ergonomic programs, organizational work strategies, and assistive devices.

## 1. Introduction

Musculoskeletal disorders are an increasingly important issue in the workplace, particularly among healthcare professionals [1]. They are characterized by damage to muscular, skeletal, and joint tissue, which ultimately impairs the proper functioning of the musculoskeletal system [2]. Work-related musculoskeletal disorders (WMSDs) result in discomfort and numerous aches and pains that affect their professional activity and quality of life at work. They are most often caused by the repetition of tasks and the assumption of awkward postures maintained for long periods.

Numerous surveys and systematic reviews have highlighted the high prevalence (>85%) of WMSDs among surgeons [3,4,5]. This high prevalence is linked to the specificities of their practice, particularly during operations which involve a heavy mental and physical workload over long periods. The need for a high level of precision [6] and concentration [7], the maintenance of static [8] standing postures, induces significant fatigue, which in the long term favors the appearance of WMSDs, and reduces productivity and work [9] quality. Several studies have investigated which body area is most affected by WMSD. Thus, Adams et al., 2013 [10] and Dabholkar et al., 2015 [4] showed that the lower back presented the greatest prevalence (75.6% and 49.3%, respectively). However, other authors have shown that the greatest prevalence has been identified for the neck [3,11], shoulder [12], or wrist [13,14]. Some authors have associated high prevalence in specific body areas with pathologies. Thus, Auerbach et al., 2011 [15] showed that orthopedic surgeons had degeneration of the cervical and lumbar spine, Mal and Costello [16] pointed out that otorhinolaryngology surgeons suffered from rotator cuff pathologies, and Capone et al., 2010 [17], reported that plastic surgeons experienced carpal tunnel syndrome. In contrast, body areas of the lower limb were less affected, with a prevalence below 10%, as reported for the hip [18], knee [19], and ankle [20], as well as the elbow [21]. However, a wide disparity in prevalence has also been observed in the literature for a single body area. Indeed, multiplier ratios of 5 to 20 can be found for the neck (7.7% [22] vs. 78.9% [11]), shoulder (8.0% [23] vs. 80.5% [12]), wrist (2.3% [24] vs. 69.5% [12]), or ankle (4.7% [25] vs. 21.0% [26]). This wide heterogeneity could be explained by geographical, methodological, demographic, sociological, psychological, or environmental differences. In this context, authors have tried to examine the effect of some of these parameters on prevalence among nurses. By studying the effect of a country’s level of development [27], age, or experience [28], the authors have provided details of the WMSD risk. To the best of our knowledge, the only studies that have attempted to exploit these parameters in surgeons are those by Gorce et al. [29]. The authors studied the effect of the continent on the WMSD prevalence in nine body areas, but only for robotic/video-assisted surgery. The results reported by these different studies show the importance of adapting health promotion intervention programs to the characteristics of healthcare professionals. It is therefore necessary to continue collecting data on the prevalence of WMSDs among surgeons worldwide.

The aim of this meta-analysis was to map the prevalence of WMSDs among surgeons by continent. Analyses were conducted for overall prevalence and prevalence for nine body areas. Because many parameters can affect the prevalence of WMSD, including changes in working conditions over time and intrinsic surgeon characteristics, a meta-regression was added. Correlations between age and year of experience as a surgeon and year of publication by continent were considered. The results will contribute to the formulation of recommendations for prevention campaigns to improve the quality of life and working environment of surgeons.

## 2. Materials and Methods

### 2.1. Inclusion/Exclusion Criteria

The PECO principle (P: Participants; E: Exposures; C: Comparisons; O: Outcomes; s: Study design) was selected to define the inclusion criteria: (1) participants: surgeons from all specialties worldwide; (2) exposures: surgeons exposed to WMSDs; (3) comparisons: prevalence of WMSDs obtained on each continent; (4) outcomes: prevalence of WMSDs, overall and by body area. According to international literature, nine body areas were assessed: neck, upper back, lower back, shoulders, elbows, wrists, hips, knees, and ankles. WMSDs were defined as “symptoms such as pain and discomfort lasting at least one week or occurring at least once a month over the past 12 months” [30]; (5) study design: cross-sectional study.

The following exclusion criteria were applied: (1) the work was not a cross-sectional study; (2) the study was not written in English; (3) it was not a peer-reviewed article; (4) the sample was a mixed population, and results were not presented by profession but for the whole group, i.e., impossible to extract results for surgeons alone; (5) insufficient details on body area prevalence (e.g., prevalence for upper or lower limb).

### 2.2. Search Strategy

The literature review was conducted in January and February 2025 in 3 open-access databases: ScienceDirect, PubMed/Medline, and Google Scholar. The following combination of keywords was used to scan each database without publication date restriction until 31 December 2024: (“musculoskeletal disorders” OR “musculoskeletal injury”) AND surgeon AND prevalence.

All the results obtained in each database were saved in a single Excel table. The duplicate removal function was applied to all titles found to remove multiple copies of the same article. Then, two reviewers separately evaluated each title and abstract to identify relevant articles according to the inclusion/exclusion criteria. The lists of articles selected by each reviewer were compared, and discrepancies were resolved by consensus. The full text of each remaining entry was retrieved and evaluated separately by the two reviewers to produce the retained article list. Any articles not meeting the inclusion/exclusion criteria were excluded. The selections of each reviewer were compared, and any discrepancies were resolved by consensus after a further review of the article to establish the final list of articles included in the analysis.

### 2.3. Quality Assessment

The quality of each included article was assessed using AXIS, a tool for evaluating cross-sectional studies [31]. The AXIS tool assesses the risk of bias based on 20 questions relating to the various aspects of a cross-sectional study. Each question is answered positively (“yes”) or negatively (“no”). The number of positive and negative responses was counted and used to define the level of risk of bias. Applying the classification proposed by Hermanson and Choi [32], the risk of bias is low if at least 80% of responses are positive; the risk is medium if the number of positive responses is between 50% and 80%; the risk is high if the number of positive responses is less than 50%. The evaluation was carried out separately by the two reviewers, and the results were compared for each study. Any discrepancies were discussed to decide on the final assessment.

### 2.4. Data Extraction

For each included article, the following demographic parameters were recorded separately by two reviewers: first author name, year of publication, country, continent, number of participants, response rate, male/female distribution, average age, height, weight, body mass index (BMI), number of years’ experience as surgeon. Total prevalence and prevalence for each of the nine body areas were then extracted for analysis by continent. All demographic and prevalence data were compiled in two summary tables (empty boxes indicate not reported data). Particular attention was paid to how prevalence was reported in each study. When a prevalence was presented in relation to the subgroup (e.g., surgeons suffering from WMSDs), it was recalculated in relation to the total sample to enable comparison with other studies. Data were compared, and any discrepancies were resolved by consensus.

### 2.5. Statistical Analysis

The method published by Neyeloff et al. [33] was used to perform the meta-analysis. Cochran’s Q test (significance level < 10%) and I^2^ statistic were used to assess the heterogeneity. The intervals proposed in Cochrane Training were retained [34]: I^2^ < 40% corresponds to low heterogeneity, I^2^ between 30% and 60% corresponds to moderate heterogeneity, I^2^ between 50% and 90% corresponds to substantial heterogeneity, and high heterogeneity was considered for I^2^ > 75%. The model choice was defined as follows: for p(Q) ≥ 0.1 and I^2^ ≤ 50%, a fixed-effects model was selected for the meta-analysis (no heterogeneity was evident among studies). Alternatively, if p(Q) < 0.1 and I^2^ > 50%, a significant heterogeneity was considered, and a random-effects model was used. A subgroup analysis was carried out to study the effect of continents on overall and body area prevalence among worldwide surgeons. A meta-regression by continent was performed to study the correlations between year of publication, age, and experience of surgeons, and the prevalence of WMSD by body area.

### 2.6. Registration and Report

The entire protocol was registered in the PROSPERO database (CRD420251010734), and the Preferred Reporting Items for Systematic reviews and Meta-Analyses (PRISMA) guidelines [35] were followed to conduct the present systematic review and report the results.

## 3. Results

### 3.1. Search Results

**Figure 1:**
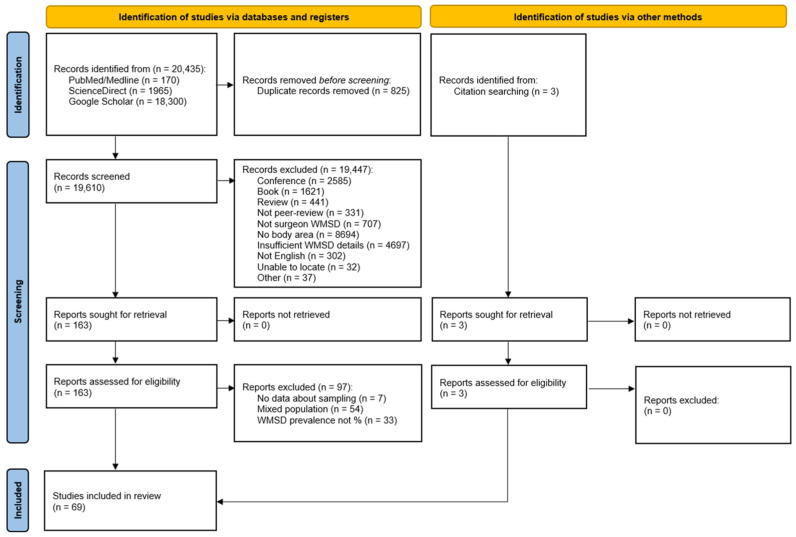
PRISMA flow diagram.

The search in all three databases identified 20,486 items. The Excel function found and removed 825 duplicates. Among the 19,661 unique items, 19,499 were excluded according to the inclusion/exclusion criteria. Full-text assessment of the remaining 162 articles led to the exclusion of 97, mainly because of the structure of the sample or the format of the reported results. An analysis of the references led to the addition of 3 further articles to the 65 selected works. Thus, the present study included 68 articles with a total of 17,188 surgeons. Figure 1 illustrates the complete selection process.

### 3.2. Study Characteristics

Table 1 displays the geographical and demographic data for each study by continent. Four studies (2 in America [20,36] and 2 in Asia [19,37]) presented information for two distinct groups of surgeons. Data for each group are reported separately in the table. The 68 studies selected were divided between three of the five continents: 36 studies in America, 15 in Asia, and 16 in Europe. In the American continent, only two countries were represented, with the largest number of studies carried out in the USA (33) [3,7,10,13,14,15,17,18,20,22,36,38,39,40,41,42,43,44,45,46,47,48,49,50,51,52,53,54,55,56,57,58,59] and only three in Canada [60,61,62]. Asian studies were conducted in seven different countries: China [63,64], India [4,19,23,24,65], Iran [66,67,68], Japan [69], Pakistan [70], Saudi Arabia [37,70], and Singapore [26]. In Europe, nine countries were involved: Germany [71], Ireland [72], Italy [11,73,74], Romania [25], Spain [75], Sweden [21], the Netherlands [76,77,78], Turkey [79], and the United Kingdom [12,16,80,81]. There was only one study from Oceania (Australia [82]), and no work from Africa met the inclusion criteria.

The sample sizes tested varied widely. A third of the cross-sectional studies examined a sample of surgeons of under 100, with a minimum of 17 [43,74]. For the others, the samples were larger, with a maximum sample size of 1086 surgeons [20]. Among the 56 samples described, 48 were mainly male, including two studies involving only males [47,69]. For the other eight, females were in the majority (with one study involving only female surgeons [14]). Across all studies, the average distribution was 74.8% males and 25.2% females. This distribution was observed in America and Asia. In Europe, the distribution was 2/3 male and 1/3 female. The mean age of the surgeons ranged from 29.5 [54] to 54 [15] years, with an average across all studies of 46.7 ± 5.0 years. The oldest population was identified in America (48.1 ± 4.1 years) and the youngest in Asia (40.4 ± 4.4 years). Worldwide experience as a surgeon averaged 14.3 ± 4.0 years. American surgeons were the most experienced (14.8 ± 3.9 years), followed by Europeans (13.6 ± 4.3 years) and Asians (10.9 ± 2.9 years). Workload was reported in half the studies in two distinct ways: number of cases treated or number of hours of practice per week (6.9 ± 5.3 cases and 11.3 ± 5.9 h on average, respectively). The number of cases treated was highest in Asia (10.4 ± 6.2 cases), while the highest number of practice hours was recorded in America (12.3 ± 6.3 h).

Table 2 summarizes the WMSD prevalence reported in each survey. Five studies investigated only one body area, and eighteen studies investigated two or three. More than half the studies investigated prevalence in at least five zones, with eleven reporting prevalence for all nine body areas studied. Only 48 studies reported the overall prevalence of WMSDs. The most studied area was the neck with 64 values, followed by the shoulder and wrist with 57 values each. The lower limb areas were the least studied, with 15, 29, and 24 values reported for hip, knee, and ankle, respectively. Prevalence by body area was investigated via a meta-analysis in the following section.

### 3.3. Quality Appraisal

The results of the quality appraisal of each of the 68 articles included are presented in Table 3. As question 14 of the AXIS tool was not applicable to the studies, the total score was set at 19. Among the 68 studies, 61 had a low risk of bias and 7 a medium risk.

### 3.4. Meta-Analysis of Overall WMSD Prevalence

**Figure 2:**
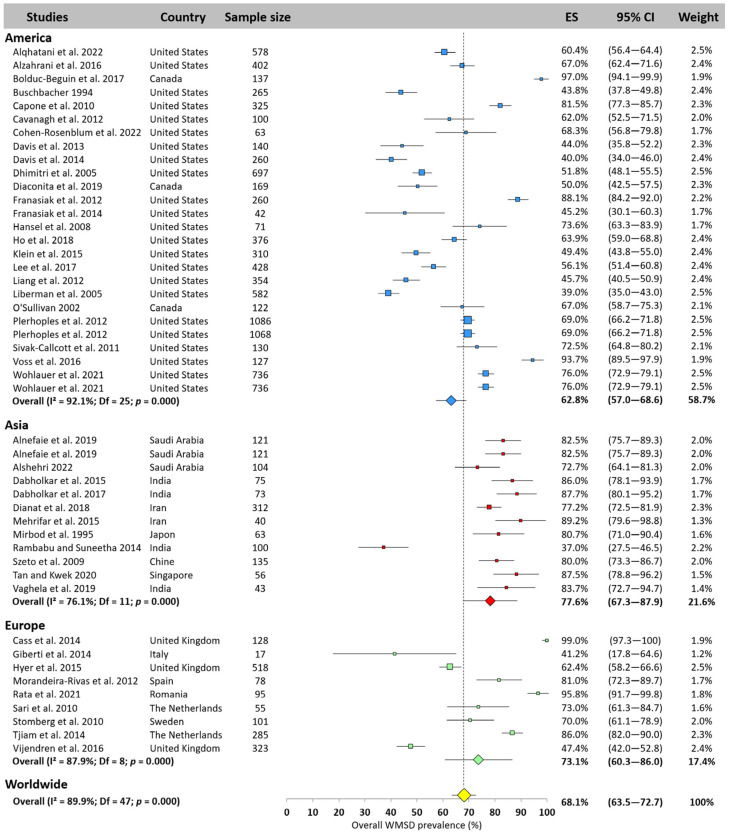
Overall WMSD prevalence pooled for America (blue), Asia (red), and Europe (green). Each square represents the mean value reported for each study. The size is proportional to the sample size tested. The horizontal line represents the 95% confidence interval. Each diamond represents the average prevalence across the continent. The yellow diamond at the bottom of the figure represents the worldwide prevalence pooled across all continents. ES = prevalence of for each study; 95% CI = 95% confidence interval. Reference: [3,4,12,14,17,18,20,26,36,37,38,39,40,41,42,45,46,49,50,53,55,56,57,58,60,62,64,65,66,67,69,70,74,75,77,78,80,81].

Figure 2 displays the overall worldwide prevalence derived from the 48 values available in the included studies, and by continent. Overall global prevalence was pooled at 68.1% (95% CI: 63.5–72.7%). A higher prevalence was observed for Asia (77.6%, 95% CI: 67.3–87.9%) and Europe (73.1%, 95% CI: 60.3–86.0%). America’s prevalence was 5% lower than the worldwide average (62.8%, 95% CI: 57.0–68.6%).

### 3.5. Meta-Analysis WMSD Prevalence by Body Area

Worldwide meta-analysis by body area showed that the three most exposed areas to WMSD were the neck and lower back, with a prevalence over 40%, followed by the shoulder (32.8%). The neck appeared most affected in America and Europe. The lower back and shoulder ranked second and third, but in a different order for the two continents. In Asia, the ranking is different. The highest prevalence was observed for the lower back, followed by the neck and upper back. The upper back and wrist ranked next, with prevalence between 20% and 30%. Lower limb prevalence was pooled at less than 20%. The hip was the least exposed area, with elbow prevalence at around 10%. Table 4 shows the ranking of continents by body area, and Table 5 displays the ranking of body areas by continent.

#### 3.5.1. Neck

**Figure 3:**
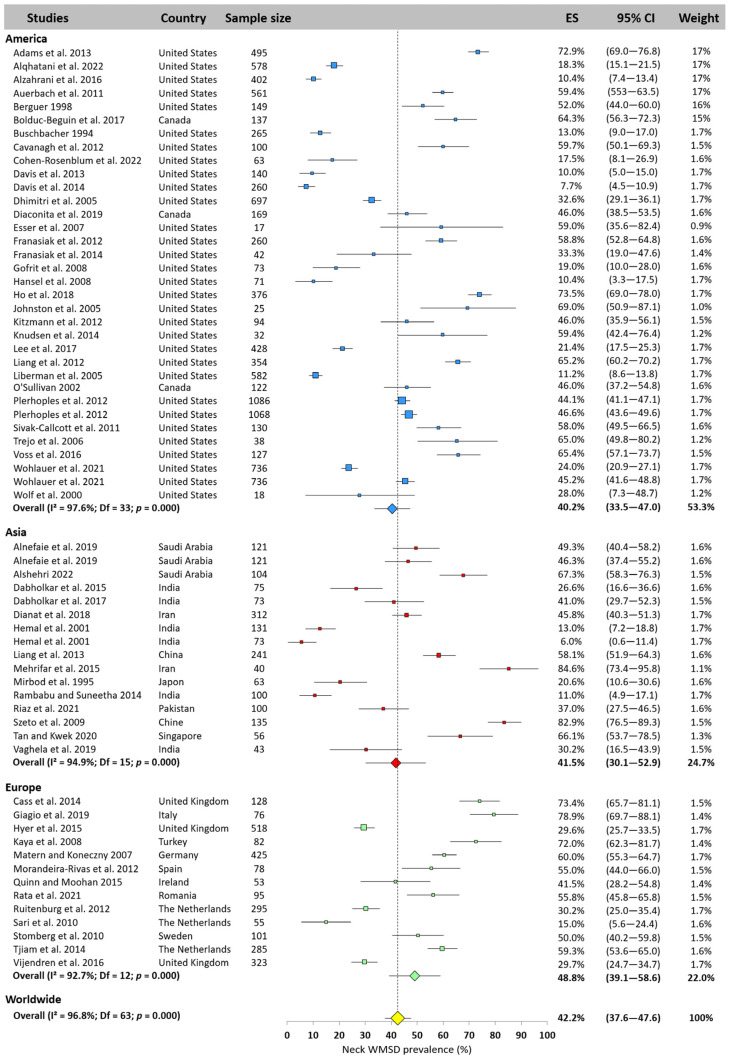
Neck WMSD prevalence pooled for America (blue), Asia (red), and Europe (green). Each square represents the mean value reported for each study. The size is proportional to the sample size tested. The horizontal line represents the 95% confidence interval. Blue, red, and green diamonds represent the average prevalence for each continent. The yellow diamond at the bottom of the figure represents the worldwide prevalence pooled across all continents. ES = prevalence for each study; 95% CI = 95% confidence interval. Reference: [3,4,7,10,11,12,13,14,15,18,19,20,21,22,23,24,25,26,36,37,38,39,40,41,42,43,45,46,47,49,50,51,52,54,55,56,57,58,59,60,61,62,63,64,65,66,67,69,70,71,72,75,76,77,78,79,80,81,83,84].

The neck was the most studied area, with 64 values extracted from the included articles. The meta-analysis showed that the neck was the area most exposed to WMSD for surgeons worldwide, with a prevalence of 42.2% (95% CI: 37.6–47.6). Prevalence in America (40.2%, 95% CI: 33.5–47.0%) and Asia (41.5%, 95% CI: 30.1–52.9%) was comparable but lower than in Europe (48.8%, 95% CI: 39.1–58.6%). Figure 3 illustrates the values for each study by continent. A high degree of heterogeneity was observed (prevalence ranging from 6.0% [19] to 84.6% [67].

#### 3.5.2. Upper Back

**Figure 4:**
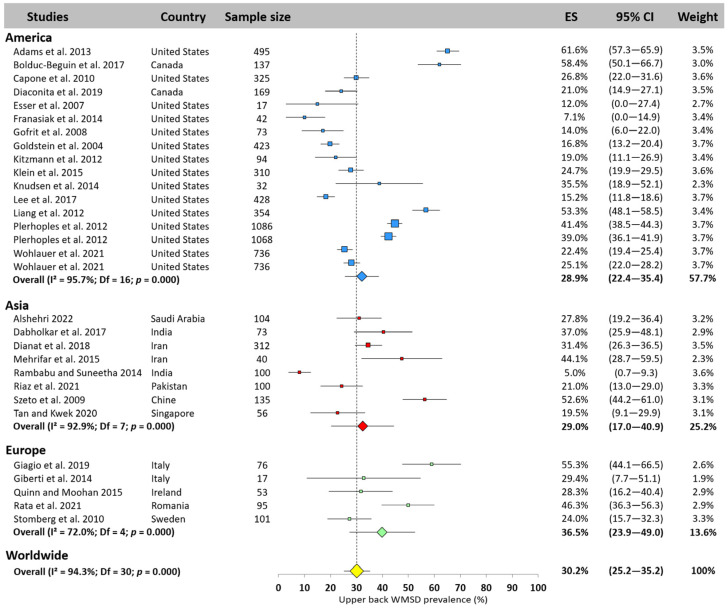
Upper back WMSD prevalence pooled for America (blue), Asia (red), and Europe (green). Each square represents the mean value reported for each study. The size is proportional to the sample size tested. The horizontal line represents the 95% confidence interval. Blue, red, and green diamonds represent the average prevalence for each continent. The yellow diamond at the bottom of the figure represents the worldwide prevalence pooled across all continents. ES = prevalence for each study; 95% CI = 95% confidence interval. Reference: [10,11,17,20,21,23,25,26,36,43,46,47,48,52,53,54,55,56,60,61,64,65,66,67,70,72,74,83].

Only 31 values were extracted from all the studies, ranging between 5.0% [23] and 61.6% [10]. The worldwide upper back WMSD prevalence was 30.2% (95% CI: 25.2–35.2%). As for neck, the values observed in America (28.9%, 95% CI: 22.4–35.4%) and Asia (29.0%, 95% CI: 17.0–40.9%) were close to the worldwide prevalence, while the prevalence observed in Europe was higher (36.5%, 95% CI: 23.9–49.0%, Figure 4).

#### 3.5.3. Lower Back

**Figure 5:**
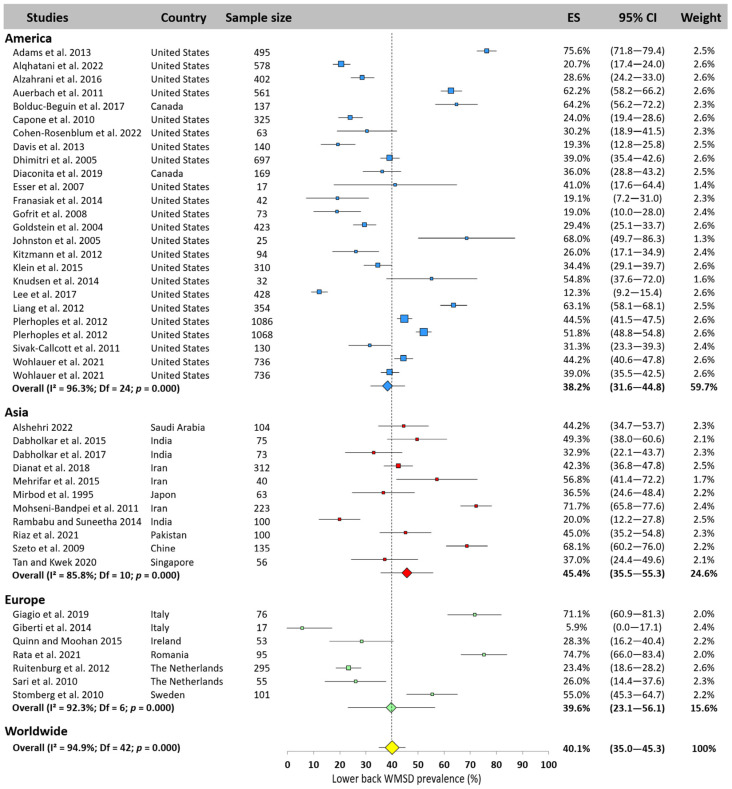
Lower back WMSD prevalence pooled for America (blue), Asia (red), and Europe (green). Each square represents the mean value reported for each study. The size is proportional to the sample size tested. The horizontal line represents the 95% confidence interval. Blue, red, and green diamonds represent the average prevalence for each continent. The yellow diamond at the bottom of the figure represents the worldwide prevalence pooled across all continents. ES = prevalence for each study; 95% CI = 95% confidence interval. Reference: [4,10,11,14,15,17,18,20,21,23,25,26,36,38,41,42,43,46,47,48,51,52,53,54,55,56,58,60,61,64,65,66,67,68,69,70,72,74,76,77,83].

The lower back ranks second in the worldwide ranking of most affected areas, with a prevalence of 40.1% (95% CI: 35.0–45.3%) based on 43 studies. The highest prevalence was observed in Asia with 45.4% (95% CI: 35.5–55.3%). Prevalence in America and Europe was lower (38.2% and 39.6%, respectively, Figure 5), closer to the worldwide prevalence. High heterogeneity was also observed between studies, with values ranging from 5.9% [74] to 75.6% [10].

#### 3.5.4. Shoulder

**Figure 6:**
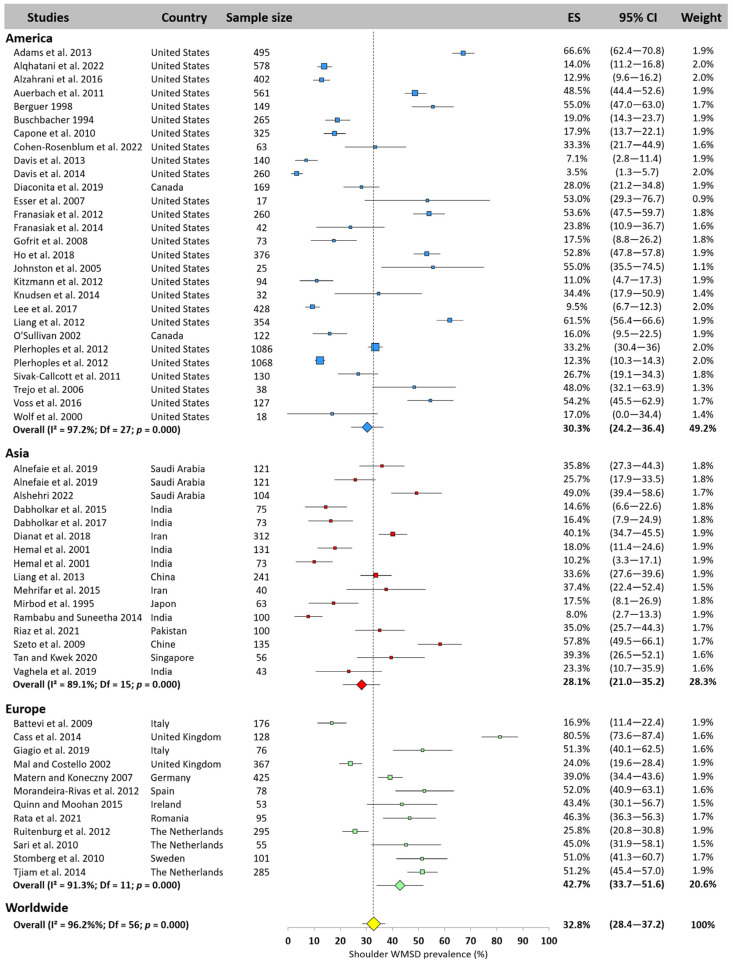
Shoulder WMSD prevalence pooled for America (blue), Asia (red), and Europe (green). Each square represents the mean value reported for each study. The size is proportional to the sample size tested. The horizontal line represents the 95% confidence interval. Blue, red, and green diamonds represent the average prevalence for each continent. The yellow diamond at the bottom of the figure represents the worldwide prevalence pooled across all continents. ES = prevalence for each study; 95% CI = 95% confidence interval. Reference: [3,4,7,10,11,12,13,14,15,16,17,18,19,20,21,22,23,24,25,26,37,38,39,41,43,45,46,47,50,51,52,54,55,56,58,59,61,62,63,64,65,66,67,68,69,70,71,72,73,75,76,77,78,83].

The shoulder was the second most studied area (57 studies) and the third most exposed to WMSD among surgeons worldwide, with a prevalence of 32.8% (95% CI: 28.4–37.2%). The lowest prevalence was observed in Asia (28.1%, 95% CI: 21.0–35.2%), followed by America (30.3%, 95% CI: 24.2–36.4%), while prevalence in Europe was over 10% higher (42.7%, 95% CI: 33.7–51.6%). Some studies reported very low prevalence (3.5% [22]), others very high (80.5% [12]). Figure 6 depicts all the prevalence reported by the studies.

#### 3.5.5. Elbow

**Figure 7:**
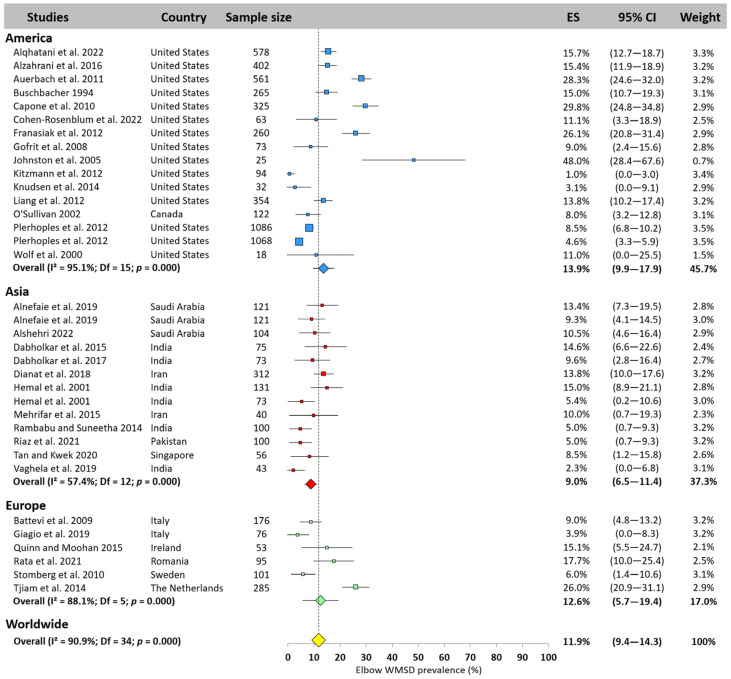
Elbow WMSD prevalence pooled for America (blue), Asia (red), and Europe (green). Each square represents the mean value reported for each study. The size is proportional to the sample size tested. The horizontal line represents the 95% confidence interval. Blue, red, and green diamonds represent the average prevalence for each continent. The yellow diamond at the bottom of the figure represents the worldwide prevalence pooled across all continents. ES = prevalence for each study; 95% CI = 95% confidence interval. Reference: [4,11,13,14,15,17,18,19,20,21,23,24,25,26,37,38,39,45,47,51,52,54,56,62,65,66,67,70,72,73,78,83].

The results showed that the elbow was the second least affected area of the body, with a worldwide prevalence of 11.9% (95% CI: 9.4–14.3%, Figure 7), pooled from 35 studies. The highest prevalence was found in America (13.9%, 95% CI: 9.9–17.9%), followed by Europe (12.6%, 95% CI: 5.7–14.3%) and Asia (9.0%, 95% CI: 6.5–11.4%). Despite this low prevalence, some authors have reported a prevalence close to 50% [51], while others have near zero [24,52].

#### 3.5.6. Wrist

**Figure 8:**
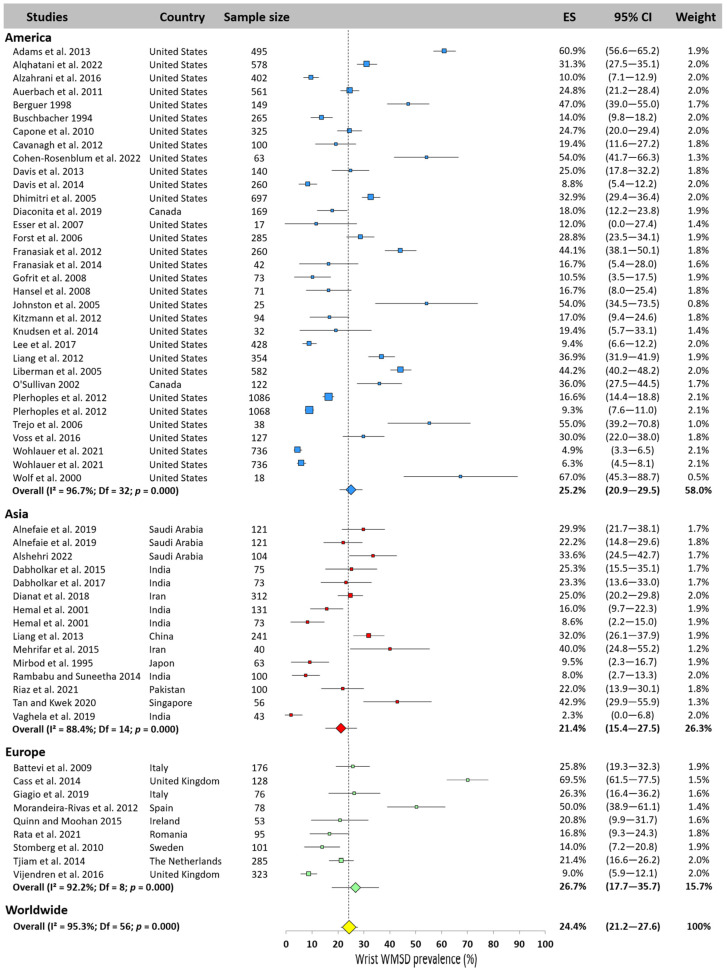
Wrist WMSD prevalence pooled for America (blue), Asia (red), and Europe (green). Each square represents the mean value reported for each study. The size is proportional to the sample size tested. The horizontal line represents the 95% confidence interval. Blue, red, and green diamonds represent the average prevalence for each continent. The yellow diamond at the bottom of the figure represents the worldwide prevalence pooled across all continents. ES = prevalence for each study; 95% CI = 95% confidence interval. Reference: [3,4,7,10,11,12,13,14,15,17,18,19,20,21,22,23,24,25,26,36,37,38,39,40,41,42,43,44,45,46,47,49,51,52,54,55,56,57,59,61,62,63,65,66,67,69,70,72,73,75,78,81,83].

Worldwide wrist prevalence was pooled at 24.4% based on 57 studies, with values ranging from 2.3% [24] to 69.5% [12]. Asia had the lowest prevalence, 21.4% (95% CI: 15.4–27.5%), while slightly higher values were found for America (25.2%, 95% CI: 20.9–29.5%) and Europe (26.7%, 95% CI: 17.7–35.7%, Figure 8).

#### 3.5.7. Hip

**Figure 9:**
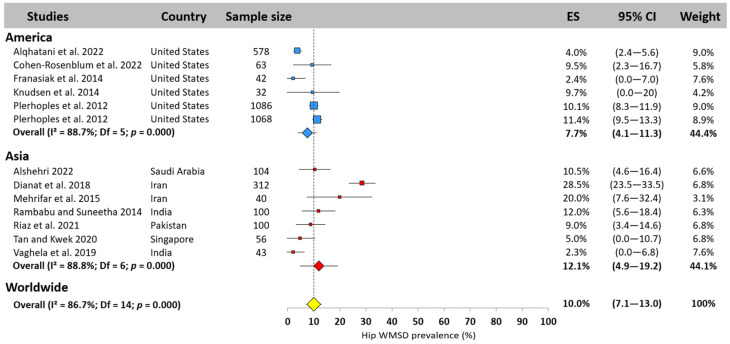
Hip WMSD prevalence pooled for America (blue) and Asia (red). Each square represents the mean value reported for each study. The size is proportional to the sample size tested. The horizontal line represents the 95% confidence interval. Blue and red diamonds represent the average prevalence for each continent The yellow diamond at the bottom of the figure represents the worldwide prevalence pooled across all continents. ES = prevalence for each study; 95% CI = 95% confidence interval. Reference: [14,18,20,23,24,26,46,54,66,67,70,83].

The hip was the least studied body area, and the one least affected by WMSDs (Figure 9). Worldwide prevalence was 10.0% (95% CI: 7.1–13.0%) based on 15 studies. American surgeons were the least exposed, with a prevalence of 7.7% (95% CI: 4.1–11.3%), in contrast to Asia, where the prevalence was 12.1% (95% CI: 4.9–19.2%). Due to the lack of European studies on this area, it was not possible to compute a prevalence for this continent. A lower heterogeneity, but with an amplitude of 25%, was observed (values between 2.3% [24] and 28.5% [66]).

#### 3.5.8. Knee

**Figure 10:**
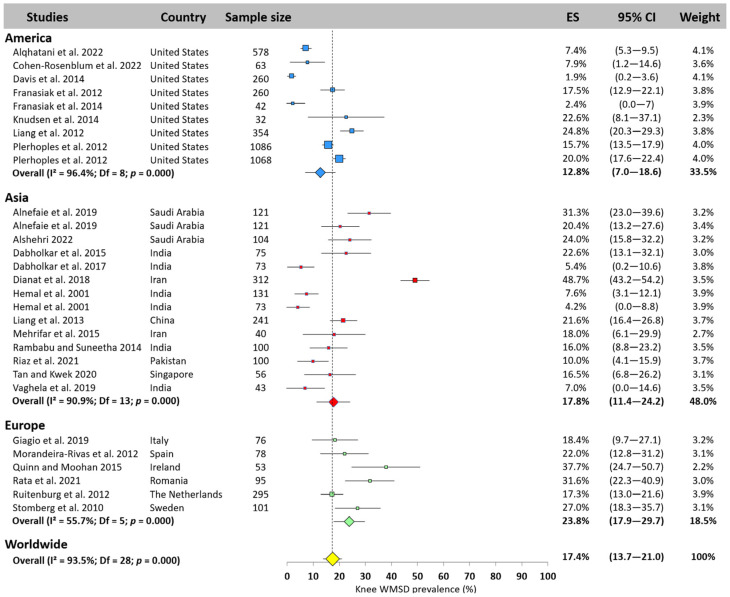
Knee WMSD prevalence pooled for America (blue), Asia (red), and Europe (green). Each square represents the mean value reported for each study. The size is proportional to the sample size tested. The horizontal line represents the 95% confidence interval. Blue, red, and green diamonds represent the average prevalence for each continent. The yellow diamond at the bottom of the figure represents the worldwide prevalence pooled across all continents. ES = prevalence for each study; 95% CI = 95% confidence interval. Reference: [4,11,14,18,19,20,21,22,23,24,25,26,37,45,46,54,56,63,65,66,67,70,72,75,76,83].

The meta-analysis revealed that the knee was the most affected area of the lower limb, but with a worldwide prevalence of only 17.4% (95% CI: 13.7–21.0%) based on 29 studies (Figure 10). Asia showed a prevalence equal to the worldwide prevalence (17.8%, 95% CI: 11.4–24.2%), America 5% lower (12.8%, 95% CI: 7.0–18.6%), and Europe 5% higher (23.8%, 95% CI: 17.9–29.7%). The prevalence reported in the studies ranged from 1.9% [22] and 48.7% [66].

#### 3.5.9. Ankle

**Figure 11:**
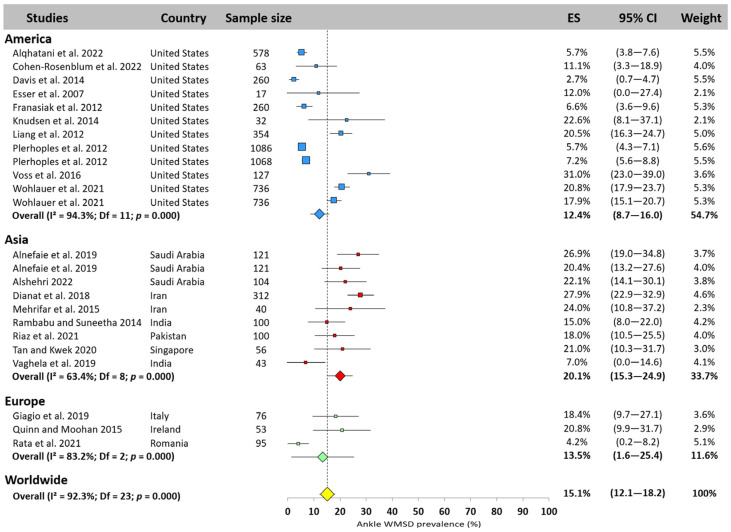
Ankle WMSD prevalence pooled for America (blue), Asia (red), and Europe (green). Each square represents the mean value reported for each study. The size is proportional to the sample size tested. The horizontal line represents the 95% confidence interval. Blue, red, and green diamonds represent the average prevalence for each continent. The yellow diamond at the bottom of the figure represents the worldwide prevalence pooled across all continents. ES = prevalence for each study; 95% CI = 95% confidence interval. Reference: [3,11,14,18,20,22,23,24,25,26,36,37,43,45,54,56,66,67,70,72,83].

The worldwide WMSD ankle prevalence among surgeons was 15.1% (95% CI: 12.1–18.2%, Figure 11). Asia ranked first, with a prevalence of 20.1% (95% CI: 15.3–24.9%), while America and Europe reported similar results, slightly lower than the worldwide prevalence (12.4%, 95% CI: 8.7–16.0% and 13.5%, 95% CI: 1.6–25.4%, respectively). Among the 24 studies that examined the ankle, some authors reported a prevalence of less than 5% [22,25], while others found values over 30% [3].

### 3.6. Meta-Regression

**Figure 12:**
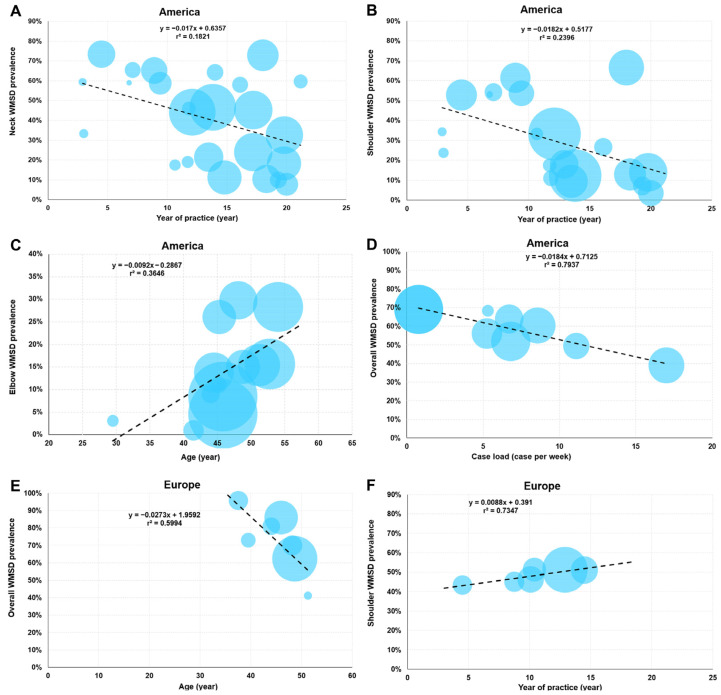
Meta-regression depicting relationships between demographic and occupational parameters and WMSD prevalence. Graphs (**A**–**D**) show the regressions for America, and graphs (**E**,**F**) display the regressions for Europe. Each blue circle represents one study. The circle size is proportional to the sample size studied in each survey.

A meta-regression was performed to study the effect of year of publication, age, and year of experience as a surgeon on the WMSD prevalence by continent. Six significant correlations were evidenced: four in America and two in Europe (Figure 12). In America, three negative correlations were found between the neck and shoulder WMSD prevalence and years of experience (r^2^ = 0.182, *p* < 0.05, and r^2^ = 0.240, *p* < 0.05, respectively), as well as between the number of cases treated per week and overall prevalence (r^2^ = 0.794, *p* < 0.05). It appears that as experience or the number of cases treated increases, the associated prevalence decreases. A positive correlation was observed between the elbow WMSD prevalence and surgeons’ age (r^2^ = 0.365, *p* < 0.05). The prevalence increased with age. In Europe, a negative correlation was highlighted between the overall prevalence of WMSD and age (r^2^ = 0.599, *p* < 0.05). Finally, a positive correlation was identified between the shoulder WMSD prevalence and years of practice as a surgeon (r^2^ = 0.735, *p* < 0.05). No correlation was observed between the year of publication and the prevalence of WMSD overall and by body area for different continents.

## 4. Discussion

The aim of the present study was to carry out a meta-analysis and meta-regression to thoroughly assess the WMSD prevalence among surgeons on each continent. Ten analyses of WMSD prevalence were conducted: overall prevalence and prevalence for each of the nine body areas reported in the literature. A review of three international open-access databases identified 68 studies that matched the selection criteria, involving 17,188 surgeons from all specialties.

### 4.1. WMSD Prevalence—Overall and by Body Area

The overall prevalence of WMSD among surgeons was pooled at 68.1%. Some meta-analyses have been conducted on the overall prevalence. Daruwalla et al. [82] (56 articles and 13,628 surgeons) reported a prevalence of 71.1% among surgeons worldwide. Vasireddi et al. [85] reported a prevalence of 73.8% (19 studies and 2974 participants), but only among orthopedic surgeons. Alleblas et al. [86] (35 publications and 7112 participants) found a prevalence of 74.0% among laparoscopic surgeons performing minimally invasive surgery. As mentioned by Daruwalla et al. [82], surgeon specialty seems to have a significant impact on the overall WMSD prevalence. In comparison with other healthcare professionals, this prevalence appears lower. Indeed, Sun et al. [27] reported an overall prevalence of 77.2% among nurses, Viera et al. [87] 90% among physiotherapists, and Chenna et al. [88] 82% among dentists. On a continental scale, the most exposed practitioners were Asian surgeons. The pooled prevalence was 77.6%, compared with 73.1% for Europe and 62.8% for American surgeons, who were significantly less exposed. The origins of these differences are multifactorial. They may result from a country’s level of development, the level of equipment available to surgeons, the work environment (ergonomic or not), the number of employees, the weekly workload, etc. To date, the limited number of studies on these criteria does not allow any conclusions to be drawn about the origins of WMSDs. However, it is worth noting the large number of studies carried out in America. Bibliometric analyses have shown that the authors and research structures that have contributed most to the study of WMSDs in surgeons were in the USA [89,90]. This result is echoed in the distribution of work included in the present study. Twice as many works were recorded in America (36 studies) as in Asia and Europe (15 and 16 studies, respectively). This shows the determination of the American continent (especially the USA) to prevent the emergence of WMSD by organizing recurrent awareness-raising and educational campaigns for surgeons [91]. Further research is needed to characterize the differences observed between continents.

Regarding the analysis by body area, the meta-analysis showed that for more than half (neck, upper back, shoulder, wrist, knee), European surgeons reported the highest prevalence. Prevalence was highest in Asia for three body areas (lower back, hip, and ankle), and highest in America for the elbow only. Differences of 8% or more were observed between the most and least exposed continents. Only the elbow and wrist showed closer prevalence between continents. These results generalize those presented by Gorce et al. [29], who had already demonstrated differences between continents, but only for assisted surgery. The classification by continent shows that the greatest prevalence observed is on the upper half of the body, whatever the continent. However, the most exposed areas vary. The top three were identical between Europe and America, i.e., neck, lower back, shoulder, while in Asia, the back (upper and lower) and neck were the most affected areas. Similar results were found in meta-analyses conducted among different groups of surgeons. The authors agree that the neck, back, and shoulder are the body parts most at risk among surgeons, but with rankings and prevalence rates differing according to specialty and continent. Thus, Daruwalla et al. [82] (56 studies for 13,628 surgeons) and Gorce et al. [29] (35 studies, 6519 participants) proposed the same classification, i.e., back (30.4% and 49.1%, respectively), neck (26.2% and 45.6%, respectively), shoulder (16.0% and 41.6%, respectively). Vasareddi et al. [85] (19 studies and 2974 participants) identified back (36.5%), neck (35.0%), and wrist (29.6%). Finally, Gorce et al. [5] (77 studies 18,952 surgeons) obtained a different ranking for assisted, i.e., back (49.9%), neck (45.3%), shoulder (41.1%) or unassisted surgery, i.e., neck (41.0%), lower back (40.0%), back (37.7%). These disparities in the body area ranking according to WMSD prevalence reinforce the observations made with regard to overall prevalence. Nevertheless, there is a consensus that the neck and lower back are the most exposed body areas, while the lower limb is much less affected by WMSD among surgeons. This observation is echoed by other healthcare professionals [5,92], such as physiotherapists [93], nurses [27,94], or dentists [88,95]. However, future work is needed to better understand the origins of differences between continents, whether environmental, societal, or organizational.

### 4.2. Age, Experience, and Year of Publication Effects on WMSD Prevalence

In the literature, the age of surgeons has been reported as a WMSD risk factor [96,97]. This result was found in American surgeons, but only for the elbow, one of the least exposed areas. This may be explained physiologically by physical decreases linked to the degradation of musculoskeletal functions and higher job stress with age [96]. On the other hand, an opposite relationship has been observed in Europe: overall prevalence seems to decrease with age. An important parameter in the occurrence of WMSD is the workload assumed by surgeons [98]. One possibility is that older surgeons are considerably reducing their operating time, which could explain the decrease in WMSD prevalence. However, it is difficult to validate this hypothesis, as this information is often not available and, as reported (case per week or hour per week), does not yet enable an accurate characterization of surgeons’ activity. Furthermore, within a single continent, the level of development and working conditions may differ considerably, which may have an impact on the level of prevalence observed in the studies.

With regard to years of experience, an increase in shoulder prevalence was observed with experience in Europe. This result is in line with the findings of other authors. Gadjradj et al. [99] reported a significant odds ratio of 0.27 in surgeons with less than 15 years’ experience. Michael et al. [100] found a 12% to 78% increase in prevalence between the beginning and after 15–20 years of practice. A physically and mentally demanding practice is an important risk factor for the emergence of WMSDs. In contrast, the correlations observed between neck and shoulder prevalence with years of experience and between overall prevalence and case load in America were all significantly negative. This could suggest that American surgeons are more aware of the risks and, with experience, are less subject to WMSDs [101].

No effect of the year of publication on WMSD prevalence was observed across continents. For several years, prevention and education policies have been proposed to reduce the risk of WMSDs [102,103]. Therefore, we could have expected a decrease in prevalence, which was not the case. Other parameters could compensate for the beneficial effects of the ergonomic programs developed. The number of cases to be treated, the increase in workload, the aging population, and the reduction in staff are all elements that may explain the absence of effects of the year of publication on the WMSD prevalence [104]. It would be very relevant to continue studies on the WMSD risk assessment and its development among surgeons to improve their working conditions and their quality of life.

### 4.3. Limitations and Recommendations for Future Works

This study has several limitations. The first is that the studies included are cross-sectional studies in which specialties were not always clearly defined. Depending on the specialty, the nature of the operations performed, their duration, and workload may be very different, and therefore more or less conducive to the appearance of WMSDs. In the future, it may be considered to treat WMSDs by specialty.

The second limitation is data heterogeneity. This is characterized by very different demographic parameters (age, gender, height, weight, experience, etc.), sample sizes, data collection methods (the questionnaire used), work environment (assisted or unassisted), type of procedure (open or minimally invasive surgery), etc. This crucial point is likely to be the main focus of future work. Prospective studies could be carried out using larger subgroups, both in cross-sectional studies and in meta-analyses.

The third limitation concerns the definition of prevalence. Similar to cross-sectional studies using a standardized questionnaire such as the Nordic Musculoskeletal Disorders Questionnaire [30] or a derivative whose prevalence was reported over 12 months, others did not specify the period of WMSD assessment. Thus, prevalence varied between a career, a year, or a shorter period without precise information. This situation could have a significant impact on the prevalence rates and, therefore, contribute to the heterogeneity observed in the present study. It would be relevant in future work to precisely define prevalence in cross-sectional studies to facilitate their use in meta-analyses.

The fourth limitation concerned the number of studies available for meta-analysis. Despite the 68 studies identified, their distribution by continent was very unbalanced, making it impossible to carry out the meta-analysis for Oceania and Africa. In addition, only 19 countries were involved in the present study: 1 for Oceania, 2 for America, 7 for Asia, and 9 for Europe. This suggests that the number of countries reporting on WMSD prevalence among surgeons (overall and by body area) remains limited, while the number of studies on WMSD is increasing [89,90].

A final methodological limitation may be mentioned. In the PRISMA method, the inclusion criteria adopted, in particular the choice of original research peer-reviewed articles written in English, may have led to the omission of some studies that could have completed the analysis by continent.

## 5. Conclusions

Meta-analysis by continent showed that overall prevalence was high in America (62.8%), Asia (77.6%), and Europe (73.1%). The areas most affected were the neck, upper and lower back, and shoulder, with prevalence ranging from 30 to 50% (their ranking differed according to continent). Six linear regressions were found between the prevalence of WMSD in four body areas and the age, year of experience, and caseload of surgeons in America and Europe. Future work could integrate more parameters, including subgroup analyses, to reduce data variability. To improve surgeons’ quality of work life, further research is needed to develop ergonomic programs, organizational work strategies, and assistive devices.

## Figures and Tables

**Table 1:** Geographical and demographic characteristics of the 68 included studies by continent.

Authors	Country	Surgeons per 100,000 Inhabitants	Sample Size	Male/Female (%)	Age * (Year)	Experience * (Year)	Case Load * (Case per Week)	Case Load * (h per Week)
**America**								
Adams et al., 2013 [10]	United States	54.71	495	50.3/49.7	47	18		8
Alqhatani et al., 2022 [18]	United States	54.71	578	84.8/15.2	52.8	19.78	8.57	
Alzahrani et al., 2016 [38]	United States	54.71	402	76.1/23.9	51.2	18.3		
Auerbach et al., 2011 [15]	United States	54.71	561		54		2.8	
Berguer 1998 [7]	United States	54.71	149					
Bolduc-Beguin et al., 2017 [60]	Canada	43.87	137	79.0/21.0	46	14		
Buschbacher 1994 [39]	United States	54.71	265	95.1/4.9	48.8 ± 8.6			
Capone et al., 2010 [17]	United States	54.71	325	87.1/12.9	48.1	12.9		
Cavanagh et al., 2012 [40]	United States	54.71	100	85.0/15.0	52.96 ± 8.03	21.17 ± 9.32		
Cohen-Rosenblum et al., 2022 [14]	United States	54.71	63	0.0/100.0	45.2	10.65	5.3	
Davis et al., 2013 [41]	United States	54.71	140		49.7 ± 10.7	19.3		13.8
Davis et al., 2014 [22]	United States	54.71	260	80.0/20.0		20 ± 11		19 ± 10
Dhimitri et al., 2005 [42]	United States	54.71	697	84.1/15.9	51.9	19.8	6.8	
Diaconita et al., 2019 [61]	Canada	43.87	169	68.0/32.0				
Esser et al., 2007 [43]	United States	54.71	17	70.6/29.4	39.6	6.8		24.3
Forst et al., 2006 [44]	United States	54.71	285	97.5/2.5	46.7	14.1		
Franasiak et al., 2012 [45]	United States	54.71	260	59.2/40.8	45.3	9.4		
Franasiak et al., 2014 [46]	United States	54.71	42	45.2/54.8		3		5.4
Gofrit et al., 2008 [47]	United States	54.71	73	100.0/0.0	44 ± 8.4	11.7 ± 8.4		3.1 ± 2.8
Goldstein et al., 2004 [48]	United States	54.71	423			13.8	9.8	
Hansel et al., 2008 [49]	United States	54.71	71	83.1/16.9	45.1			
Ho et al., 2018 [50]	United States	54.71	376	72.7/27.3	34.7 ± 8.69	4.48	6.7	
Johnston et al., 2005 [51]	United States	54.71	25				1.6	
Kitzmann et al., 2012 [52]	United States	54.71	94	66.0/34.0	41.5 ± 10.9	11.8		
Klein et al., 2015 [53]	United States	54.71	310	89.0/11.0	49	16	11.1	
Knudsen et al., 2014 [54]	United States	54.71	32	75.0/25.0	29.5	2.9		33.8
Lee et al., 2017 [55]	United States	54.71	428	71.3/28.7	47.61 ± 9.2	13.48 ± 9.5	5.24 ± 4.2	
Liang et al., 2012 [56]	United States	54.71	354	70.9/29.1	44.5	8.9		
Liberman et al., 2005 [57]	United States	54.71	582	89.3/10.7	48	14.8	17	
O’Sullivan 2002 [62]	Canada	43.87	122					
Plerhoples et al., 2012 [20]	United States	54.71	1086	72.7/27.3	45.8 ± 9.0	12.1 ± 8.3	0.77	
Plerhoples et al., 2012 [20]	United States	54.71	1068	72.7/27.3	45.8 ± 9.0	13.8 ± 9.2	0.77	
Sivak-Callcott et al., 2011 [58]	United States	54.71	130	85.4/14.6	48	16.1		13.8
Trejo et al., 2006 [59]	United States	54.71	38					
Voss et al., 2016 [3]	United States	54.71	127	60.6/39.4		7.1		
Wohlauer et al., 2021 [61]	United States	54.71	736	83.6/16.4	51.4 ± 10.9	17.2 ± 11.6		
Wohlauer et al., 2021 [61]	United States	54.71	736	83.6/16.4	51.4 ± 10.9	17.2 ± 11.6		
Wolf et al., 2000 [13]	United States	54.71	18		45.5			
**Total**			**11,774**	**77.4/22.6**	**48.1 ± 4.1**	**14.8 ± 3.9**	**5.2 ± 3.7**	**12.3 ± 6.3**
**Asia**								
Alnefaie et al., 2019 [37]	Saudi Arabia		121	61.2/38.8	34.38			
Alnefaie et al., 2019 [37]	Saudi Arabia		121	61.2/38.8	34.38			
Alshehri 2022 [70]	Saudi Arabia		104	73.1/26.9	43.36 ± 10.7	12.93	4.53	
Dabholkar et al., 2015 [4]	India	6.82	75	74.0/26.0	43.4	14.06	10.24	
Dabholkar et al., 2017 [65]	India	6.82	73	63.1/36.9	37.38 ± 10.79	10.60 ± 9.1	4.86 ± 2.23	
Dianat et al., 2018 [66]	Iran	5.02	312	65.1/34.9	45.2 ± 9.3	12.5 ± 8.3	13.1 ± 7.3	
Hemal et al., 2001 [19]	India	6.82	131		41.6 ± 6.1	4.8 ± 2.5		8.8 ± 4.8
Hemal et al., 2001 [19]	India	6.82	73		36.9 ± 8.4	10.9 ± 7.6		12.9 ± 5.7
Liang et al., 2013 [63]	China	40.13	241	96.7/3.3	44.3		5.28	
Mehrifar et al., 2018 [67]	Iran	5.02	40	63.0/37.0	38.54 ± 8.34	8.7		
Mirbod et al. 1995 [69]	Japan	37.36	63	100.0/0.0	41.8 ± 9.5	16.6 ± 9.5		
Mohseni-Bandpei et al., 2011 [68]	Iran	5.02	223	48.4/51.6	42.6	10.5		
Rambabu and Suneetha 2014 [23]	India	6.82	100					
Riaz et al., 2021 [83]	Pakistan	5.53	100	48.0/52.0	33.13 ± 11	7.48 ± 9.51	24.78	
Szeto et al., 2009 [64]	China	40.13	135	82.2/17.8	35.3	10.0 ± 7.3		
Tan and Kwek 2020 [26]	Singapore	31.45	56		33			16
Vaghela et al., 2019 [24]	India		6.82	43	69.8/30.2	42.07	15.14	
**Total**			**2011**	**69.5/30.5**	**40.4 ± 4.4**	**10.9 ± 2.9**	**10.4 ± 6.2**	**11.5 ± 2.9**
**Europe**								
Battevi et al., 2009 [73]	Italy	142.4	176	38.6/61.4	42.8			
Cass et al., 2014 [12]	United Kingdom	133.3	128					
Giagio et al., 2019 [11]	Italy	142.4	76	65.8/34.2	37.7 ± 12.1	10.4 ± 11.3	16.1	
Giberti et al., 2014 [74]	Italy	142.4	17	94.1/5.9	51.3	3		6.0
Hyer et al., 2015 [80]	United Kingdom	133.3	518	74.5/25.5	48.7			8.78
Kaya et al., 2008 [79]	Turkey	47.63	82					
Mal and Costello 2002 [16]	United Kingdom	133.3	367		51.2			
Matern and Koneczny 2007 [71]	Germany	108	425	75.0/25.0				
Morandeira-Rivas et al., 2012 [75]	Spain	79.9	78	87.0/13.0	44.06		1.3	
Quinn and Moohan 2015 [72]	Ireland	71.97	53	36.0/64.0	30.4	4.5		
Rata et al., 2021 [25]	Romania	60.53	95	62.1/37.9	37.56 ± 8.74	10.09 ± 8.41	7.56 ± 3.73	
Ruitenburg et al., 2012 [76]	The Netherlands	48.97	295	45.0/55.0	40.0 ± 9.8			
Sari et al., 2010 [77]	The Netherlands	48.97	55	65.4/34.6	39.5 ± 7.3	8.75 ± 6.07		6
Stomberg et al., 2010 [21]	Sweden	113.11	101	36.6/63.4	48.2 ± 10.2	14.5 ± 10.4		1.9
Tjiam et al., 2014 [78]	The Netherlands	48.97	285	93.0/7.0	46	12.9		6.45
Vijendren et al., 2016 [81]	United Kingdom	133.3	323			18.7		
**Total**			**3074**	**67.0/33.0**	**45.5 ± 5.2**	**13.6 ± 4.3**	**8.2 ± 5.8**	**7.2 ± 2.1**
**Oceania**								
Grant et al., 2020 [84]	Australia	45.11	329	73.0/27.0				20
**Worldwide total**			**17,188**	**74.8/25.2**	**46.7 ± 5.0**	**14.3 ± 4.0**	**6.9 ± 5.3**	**11.3 ± 5.9**

* Age, year of experience, and case load are presented as mean (±standard deviation when available).

**Table 2:** WMSD prevalence (%) of the 68 included studies by continent.

Authors	Country	Sample Size	WMSD Prevalence by Body Area									Overall WMSD Prevalence
			Neck	Upper Back	Lower Back	Shoulder	Elbow	Wrist	Hip	Knee	Ankle	
**America**												
Adams et al., 2013 [10]	United States	495	72.9%	61.6%	75.6%	66.6%		60.9%				
Alqhatani et al., 2022 [18]	United States	578	18.3%		20.7%	14.0%	15.7%	31.3%	4.0%	7.4%	5.7%	60.4%
Alzahrani et al., 2016 [38]	United States	402	10.4%		28.6%	12.9%	15.4%	10.0%				67.0%
Auerbach et al., 2011 [15]	United States	561	59.4%		62.2%	48.5%	28.3%	24.8%				
Berguer 1998 [7]	United States	149	52.0%			55.0%		47.0%				
Bolduc-Beguin et al., 2017 [60]	Canada	137	64.3%	58.4%	64.2%							97.0%
Buschbacher 1994 [39]	United States	265	13.0%			19.0%	15.0%	14.0%				43.8%
Capone et al., 2010 [17]	United States	325		26.8%	24.0%	17.9%	29.8%	24.7%				81.5%
Cavanagh et al., 2012 [40]	United States	100	59.7%					19.4%				62.0%
Cohen-Rosenblum et al., 2022 [14]	United States	63	17.5%		30.2%	33.3%	11.1%	54.0%	9.5%	7.9%	11.1%	68.3%
Davis et al., 2013 [41]	United States	140	10.0%		19.3%	7.1%		25.0%				44.0%
Davis et al., 2014 [22]	United States	260	7.7%			3.5%		8.8%		1.9%	2.7%	40.0%
Dhimitri et al., 2005 [42]	United States	697	32.6%		39.0%			32.9%				51.8%
Diaconita et al., 2019 [61]	Canada	169	46.0%	21.0%	36.0%	28.0%		18.0%				50.0%
Esser et al., 2007 [43]	United States	17	59.0%	12.0%	41.0%	53.0%		12.0%			12.0%	
Forst et al., 2006 [44]	United States	285						28.8%				
Franasiak et al., 2012 [45]	United States	260	58.8%			53.6%	26.1%	44.1%		17.5%	6.6%	88.1%
Franasiak et al., 2014 [46]	United States	42	33.3%	7.1%	19.1%	23.8%		16.7%	2.4%	2.4%		45.2%
Gofrit et al., 2008 [47]	United States	73	19.0%	14.0%	19.0%	17.5%	9.0%	10.5%				
Goldstein et al., 2004 [48]	United States	423		16.8%	29.4%							
Hansel et al., 2008 [49]	United States	71	10.4%					16.7%				73.6%
Ho et al., 2018 [50]	United States	376	73.5%			52.8%						63.9%
Johnston et al., 2005 [51]	United States	25	69.0%		68.0%	55.0%	48.0%	54.0%				
Kitzmann et al., 2012 [52]	United States	94	46.0%	19.0%	26.0%	11.0%	1.0%	17.0%				
Klein et al., 2015 [53]	United States	310		24.7%	34.4%							49.4%
Knudsen et al., 2014 [54]	United States	32	59.4%	35.5%	54.8%	34.4%	3.1%	19.4%	9.7%	22.6%	22.6%	
Lee et al., 2017 [55]	United States	428	21.4%	15.2%	12.3%	9.5%		9.4%				56.1%
Liang et al., 2012 [56]	United States	354	65.2%	53.3%	63.1%	61.5%	13.8%	36.9%		24.8%	20.5%	45.7%
Liberman et al., 2005 [57]	United States	582	11.2%					44.2%				39.0%
O’Sullivan 2002 [62]	Canada	122	46.0%			16.0%	8.0%	36.0%				67.0%
Plerhoples et al., 2012 [20]	United States	1086	44.1%	41.4%	44.5%	33.2%	8.5%	16.6%	10.1%	15.7%	5.7%	69.0%
Plerhoples et al., 2012 [20]	United States	1068	46.6%	39.0%	51.8%	12.3%	4.6%	9.3%	11.4%	20.0%	7.2%	69.0%
Sivak-Callcott et al., 2011 [58]	United States	130	58.0%		31.3%	26.7%						72.5%
Trejo et al., 2006 [59]	United States	38	65.0%			48.0%		55.0%				
Voss et al., 2016 [3]	United States	127	65.4%			54.2%		30.0%			31.0%	93.7%
Wohlauer et al., 2021 [61]	United States	736	24.0%	22.4%	44.2%			4.9%			20.8%	76.0%
Wohlauer et al., 2021 [61]	United States	736	45.2%	25.1%	39.0%			6.3%			17.9%	76.0%
Wolf et al., 2000 [13]	United States	18	28.0%			17.0%	11.0%	67.0%				
**Asia**												
Alnefaie et al., 2019 [37]	Saudi Arabia	121	49.3%			35.8%	13.4%	29.9%		31.3%	26.9%	82.5%
Alnefaie et al., 2019 [37]	Saudi Arabia	121	46.3%			25.7%	9.3%	22.2%		20.4%	20.4%	82.5%
Alshehri 2022 [70]	Saudi Arabia	104	67.3%	27.8%	44.2%	49.0%	10.5%	33.6%	10.5%	24.0%	22.10%	72.7%
Dabholkar et al., 2015 [4]	India	75	26.6%		49.3%	14.6%	14.6%	25.3%		22.6%		86.0%
Dabholkar et al., 2017 [65]	India	73	41.0%	37.0%	32.9%	16.4%	9.6%	23.3%		5.4%		87.7%
Dianat et al., 2018 [66]	Iran	312	45.8%	31.4%	42.3%	40.1%	13.8%	25.0%	28.5%	48.7%	27.9%	77.2%
Hemal et al., 2001 [19]	India	131	13.0%			18.0%	15.0%	16.0%		7.6%		
Hemal et al., 2001 [19]	India	73	6.0%			10.2%	5.4%	8.6%		4.2%		
Liang et al., 2013 [63]	China	241	58.1%			33.6%		32.0%		21.6%		
Mehrifar et al., 2018 [67]	Iran	40	84.6%	44.1%	56.8%	37.4%	10.0%	40.0%	20.0%	18.0%	24.0%	89.2%
Mirbod et al. 1995 [69]	Japan	63	20.6%		36.5%	17.5%		9.5%				80.7%
Mohseni-Bandpei et al., 2011 [68]	Iran	223			71.7%							
Rambabu and Suneetha 2014 [23]	India	100	11.0%	5.0%	20.0%	8.0%	5.0%	8.0%	12.0%	16.0%	15.0%	37.0%
Riaz et al., 2021 [83]	Pakistan	100	37.0%	21.0%	45.0%	35.0%	5.0%	22.0%	9.0%	10.0%	18.0%	
Szeto et al., 2009 [64]	China	135	82.9%	52.6%	68.1%	57.8%						80.0%
Tan and Kwek 2020 [26]	Singapore	56	66.1%	19.5%	37.0%	39.3%	8.5%	42.9%	5.0%	16.5%	21.0%	87.5%
Vaghela et al., 2019 [24]	India	6.82	30.2%			23.3%	2.3%	2.3%	2.3%	7.0%	7.0%	83.7%
**Europe**												
Battevi et al., 2009 [73]	Italy	176				16.9%	9.0%	25.8%				
Cass et al., 2014 [12]	United Kingdom	128	73.4%			80.5%		69.5%				99.0%
Giagio et al., 2019 [11]	Italy	76	78.9%	55.3%	71.1%	51.3%	3.9%	26.3%	14.5%	18.4%	18.4%	
Giberti et al., 2014 [74]	Italy	17		29.4%	5.9%							41.2%
Hyer et al., 2015 [80]	United Kingdom	518	29.6%									62.4%
Kaya et al., 2008 [79]	Turkey	82	72.0%									
Mal and Costello 2002 [16]	United Kingdom	367				24.0%						
Matern and Koneczny 2007 [71]	Germany	425	60.0%			39.0%						
Morandeira-Rivas et al., 2012 [75]	Spain	78	55.0%			52.0%		50.0%		22.0%		81.0%
Quinn and Moohan 2015 [72]	Ireland	53	41.5%	28.3%	28.3%	43.4%	15.1%	20.8%		37.7%	20.8%	
Rata et al., 2021 [25]	Romania	95	55.8%	46.3%	74.7%	46.3%	17.7%	16.8%	11.6%	31.6%	4.2%	95.8%
Ruitenburg et al., 2012 [76]	The Netherlands	295	30.2%		23.4%	25.8%				17.3%		
Sari et al., 2010 [77]	The Netherlands	55	15.0%		26.0%	45.0%						73.0%
Stomberg et al., 2010 [21]	Sweden	101	50.0%	24.0%	55.0%	51.0%	6.0%	14.0%		27.0%		70.0%
Tjiam et al., 2014 [78]	The Netherlands	285	59.3%			51.2%	26.0%	21.4%				86.0%
Vijendren et al., 2016 [81]	United Kingdom	323	29.7%					9.0%				47.4%
**Oceania**												
Grant et al., 2020 [84]	Australia	329	59.0%	38.0%		55.0%						75.0%

**Table 3:** Quality appraisal of each included study according to AXIS tool.

	1. Were the Aims/Objectives of the Study Clear?	2. Was the Study Design Appropriate for the Stated Aim(s)?	3. Was the Sample Size Justified?	4. Was the Target/Reference Population Clearly Defined? (Is it Clear Who the Research Was About?)	5. Was the Sample Frame Taken from An appropriate Population Base so that it Closely Represented the Target/Reference Population Under Investigation?	6. Was the Selection Process Likely to Select Subjects/Participants that Were Representative of the Target/Reference Population Under Investigation?	7. Were Measures Undertaken to Address and Categorise Non-Responders?	8. Were the Risk Factor and Outcome Variables Measured Appropriate to the Aims of the Study?	9. Were the Risk Factor and Outcome Variables Measured Correctly Using Instruments/Measurements that Had Been Trialled, Piloted or Published Previously?	10. Is it Clear What Was Used to Determined Statistical Significance and/or Precision Estimates? (e.g., *p* Values, CIs)	11. Were the Methods (Including Statistical Methods) Sufficiently Described to Enable Them to Be Repeated?	12. Were the Basic Data Adequately Described?	13. Does the Response Rate Raise Concerns About Non-Response Bias?	14. If Appropriate, Was information About Non-Responders Described?	15. Were the Results Internally Consistent?	16. Were the Results for the Analyses Described in the Methods, Presented?	17. Were the Authors’ Discussions and Conclusions Justified by the Results?	18. Were the Limitations of the Study Discussed?	19. Were There Any Funding Sources or Conflicts of Interest That May Affect the Authors’ Interpretation of The Results? *	20. Was Ethical Approval or Consent of Participants Attained?	Yes	No	Yes (%)	Risk of Biais
Adams et al., 2013 [10]	Yes	Yes	No	Yes	Yes	Yes	No	Yes	Yes	Yes	Yes	Yes	No	NA	Yes	Yes	Yes	Yes	No	Yes	16	3	89%	Low
Alnefaie et al., 2019 [37]	Yes	Yes	No	Yes	Yes	Yes	No	Yes	Yes	Yes	Yes	Yes	No	NA	Yes	Yes	Yes	No	No	Yes	15	4	84%	Low
Alqhatani et al., 2022 [18]	Yes	Yes	No	Yes	Yes	Yes	No	Yes	Yes	Yes	Yes	Yes	No	NA	Yes	Yes	Yes	Yes	No	Yes	16	3	89%	Low
Alshehri 2022 [70]	Yes	Yes	No	Yes	Yes	Yes	No	Yes	Yes	Yes	Yes	Yes	No	NA	Yes	Yes	Yes	Yes	No	Yes	16	3	89%	Low
Alzahrani et al., 2016 [38]	Yes	Yes	No	Yes	Yes	Yes	No	Yes	Yes	Yes	Yes	Yes	No	NA	Yes	Yes	Yes	Yes	No	Yes	16	3	89%	Low
Auerbach et al., 2011 [15]	Yes	Yes	No	Yes	Yes	Yes	No	Yes	Yes	Yes	Yes	Yes	No	NA	Yes	Yes	Yes	Yes	No	Yes	16	3	89%	Low
Battevi et al., 2009 [73]	Yes	Yes	No	Yes	Yes	Yes	No	Yes	Yes	No	Yes	Yes	No	NA	Yes	Yes	Yes	No	No	Yes	14	5	79%	Medium
Berguer 1998 [7]	Yes	Yes	No	Yes	Yes	Yes	No	Yes	Yes	No	Yes	Yes	No	NA	Yes	Yes	Yes	No	No	Yes	14	5	79%	Medium
Bolduc-Beguin et al., 2017 [60]	Yes	Yes	Yes	Yes	Yes	Yes	No	Yes	Yes	Yes	Yes	Yes	No	NA	Yes	Yes	Yes	No	No	Yes	16	3	89%	Low
Buschbacher 1994 [39]	Yes	Yes	Yes	Yes	Yes	Yes	No	Yes	Yes	Yes	Yes	Yes	No	NA	Yes	Yes	Yes	No	No	Yes	16	3	89%	Low
Capone et al., 2010 [17]	Yes	Yes	No	Yes	Yes	Yes	No	Yes	Yes	Yes	Yes	Yes	No	NA	Yes	Yes	Yes	Yes	No	Yes	16	3	89%	Low
Cass et al., 2014 [12]	Yes	Yes	No	Yes	Yes	Yes	No	Yes	Yes	Yes	Yes	Yes	No	NA	Yes	Yes	Yes	Yes	No	Yes	16	3	89%	Low
Cavanagh et al., 2012 [40]	Yes	Yes	No	Yes	Yes	Yes	No	Yes	Yes	Yes	Yes	Yes	No	NA	Yes	Yes	Yes	Yes	No	Yes	16	3	89%	Low
Cohen-Rosenblum et al., 2022 [14]	Yes	Yes	No	Yes	Yes	Yes	No	Yes	Yes	Yes	Yes	Yes	No	NA	Yes	Yes	Yes	Yes	No	Yes	16	3	89%	Low
Dabholkar et al., 2015 [4]	Yes	Yes	No	Yes	Yes	Yes	No	Yes	Yes	No	Yes	Yes	No	NA	Yes	Yes	Yes	Yes	No	Yes	15	4	84%	Low
Dabholkar et al., 2017 [65]	Yes	Yes	No	Yes	Yes	Yes	No	Yes	Yes	Yes	Yes	Yes	No	NA	Yes	Yes	Yes	No	No	Yes	15	4	84%	Low
Davis et al., 2013 [41]	Yes	Yes	No	Yes	Yes	Yes	No	Yes	Yes	No	Yes	Yes	No	NA	Yes	Yes	Yes	Yes	No	Yes	15	4	84%	Low
Davis et al., 2014 [22]	Yes	Yes	No	Yes	Yes	Yes	No	Yes	Yes	No	Yes	Yes	No	NA	Yes	Yes	Yes	No	No	Yes	14	5	79%	Medium
Dhimitri et al., 2005 [42]	Yes	Yes	No	Yes	Yes	Yes	No	Yes	Yes	Yes	Yes	Yes	No	NA	Yes	Yes	Yes	No	No	Yes	15	4	84%	Low
Diaconita et al., 2019 [61]	Yes	Yes	No	Yes	Yes	Yes	No	Yes	Yes	Yes	Yes	Yes	No	NA	Yes	Yes	Yes	No	No	Yes	15	4	84%	Low
Dianat et al., 2018 [66]	Yes	Yes	No	Yes	Yes	Yes	No	Yes	Yes	Yes	Yes	Yes	No	NA	Yes	Yes	Yes	Yes	No	Yes	16	3	89%	Low
Esser et al., 2007 [43]	Yes	Yes	No	Yes	Yes	Yes	No	Yes	Yes	No	Yes	Yes	No	NA	Yes	Yes	Yes	No	No	Yes	14	5	79%	Medium
Forst et al., 2006 [44]	Yes	Yes	No	Yes	Yes	Yes	No	Yes	Yes	Yes	Yes	Yes	No	NA	Yes	Yes	Yes	Yes	No	Yes	16	3	89%	Low
Franasiak et al., 2012 [45]	Yes	Yes	No	Yes	Yes	Yes	No	Yes	Yes	Yes	Yes	Yes	No	NA	Yes	Yes	Yes	Yes	No	Yes	16	3	89%	Low
Franasiak et al., 2014 [46]	Yes	Yes	No	Yes	Yes	Yes	No	Yes	Yes	No	Yes	Yes	No	NA	Yes	Yes	Yes	Yes	No	Yes	15	4	84%	Low
Giagio et al., 2019 [11]	Yes	Yes	Yes	Yes	Yes	Yes	No	Yes	Yes	Yes	Yes	Yes	No	NA	Yes	Yes	Yes	Yes	No	Yes	17	2	95%	Low
Giberti et al., 2014 [74]	Yes	Yes	No	Yes	Yes	Yes	No	Yes	Yes	Yes	Yes	Yes	No	NA	Yes	Yes	Yes	Yes	No	Yes	16	3	89%	Low
Gofrit et al., 2008 [47]	Yes	Yes	No	Yes	Yes	Yes	No	Yes	Yes	Yes	Yes	Yes	No	NA	Yes	Yes	Yes	No	No	Yes	15	4	84%	Low
Goldstein et al., 2004 [48]	Yes	Yes	No	Yes	Yes	Yes	No	Yes	Yes	No	Yes	Yes	No	NA	Yes	Yes	Yes	Yes	No	Yes	15	4	84%	Low
Grant et al., 2020 [84]	Yes	Yes	Yes	Yes	Yes	Yes	No	Yes	Yes	Yes	Yes	Yes	No	NA	Yes	Yes	Yes	Yes	No	Yes	17	2	95%	Low
Hansel et al., 2008 [49]	Yes	Yes	No	Yes	Yes	Yes	No	Yes	Yes	Yes	Yes	Yes	No	NA	Yes	Yes	Yes	Yes	No	Yes	16	3	89%	Low
Hemal et al., 2001 [19]	Yes	Yes	No	Yes	Yes	Yes	No	Yes	Yes	Yes	Yes	Yes	No	NA	Yes	Yes	Yes	No	No	Yes	15	4	84%	Low
Ho et al., 2018 [50]	Yes	Yes	No	Yes	Yes	Yes	No	Yes	Yes	Yes	Yes	Yes	No	NA	Yes	Yes	Yes	Yes	No	Yes	16	3	89%	Low
Hyer et al., 2015 [80]	Yes	Yes	No	Yes	Yes	Yes	No	Yes	Yes	Yes	Yes	Yes	No	NA	Yes	Yes	Yes	Yes	No	Yes	16	3	89%	Low
Johnston et al., 2005 [51]	Yes	Yes	No	Yes	Yes	Yes	No	Yes	Yes	No	Yes	Yes	No	NA	Yes	Yes	Yes	Yes	No	Yes	15	4	84%	Low
Kaya et al., 2008 [79]	Yes	Yes	No	Yes	Yes	Yes	No	Yes	Yes	No	Yes	Yes	No	NA	Yes	Yes	Yes	No	No	Yes	14	5	79%	Medium
Kitzmann et al., 2012 [52]	Yes	Yes	No	Yes	Yes	Yes	No	Yes	Yes	Yes	Yes	Yes	No	NA	Yes	Yes	Yes	Yes	No	Yes	16	3	89%	Low
Klein et al., 2015 [53]	Yes	Yes	No	Yes	Yes	Yes	No	Yes	Yes	Yes	Yes	Yes	No	NA	Yes	Yes	Yes	Yes	No	Yes	16	3	89%	Low
Knudsen et al., 2014 [54]	Yes	Yes	No	Yes	Yes	Yes	No	Yes	Yes	No	Yes	Yes	No	NA	Yes	Yes	Yes	Yes	No	Yes	15	4	84%	Low
Lee et al., 2017 [55]	Yes	Yes	No	Yes	Yes	Yes	No	Yes	Yes	Yes	Yes	Yes	No	NA	Yes	Yes	Yes	Yes	No	Yes	16	3	89%	Low
Liang et al., 2012 [56]	Yes	Yes	No	Yes	Yes	Yes	No	Yes	Yes	Yes	Yes	Yes	No	NA	Yes	Yes	Yes	Yes	No	Yes	16	3	89%	Low
Liang et al., 2013 [63]	Yes	Yes	No	Yes	Yes	Yes	No	Yes	Yes	Yes	Yes	Yes	No	NA	Yes	Yes	Yes	Yes	No	Yes	16	3	89%	Low
Liberman et al., 2005 [57]	Yes	Yes	No	Yes	Yes	Yes	No	Yes	Yes	Yes	Yes	Yes	No	NA	Yes	Yes	Yes	Yes	No	Yes	16	3	89%	Low
Mal and Costello 2002 [16]	Yes	Yes	No	Yes	Yes	Yes	No	Yes	Yes	Yes	Yes	Yes	No	NA	Yes	Yes	Yes	No	No	Yes	15	4	84%	Low
Matern and Koneczny 2007 [71]	Yes	Yes	No	Yes	Yes	Yes	No	Yes	Yes	No	Yes	Yes	No	NA	Yes	Yes	Yes	No	No	Yes	14	5	79%	Medium
Mehrifar et al., 2015 [67]	Yes	Yes	No	Yes	Yes	Yes	No	Yes	Yes	No	Yes	Yes	No	NA	Yes	Yes	Yes	No	No	Yes	14	5	79%	Medium
Mirbod et al. 1995 [69]	Yes	Yes	No	Yes	Yes	Yes	No	Yes	Yes	Yes	Yes	Yes	No	NA	Yes	Yes	Yes	No	No	Yes	15	4	84%	Low
Mohseni-Bandpei et al., 2011 [68]	Yes	Yes	No	Yes	Yes	Yes	No	Yes	Yes	Yes	Yes	Yes	No	NA	Yes	Yes	Yes	Yes	No	Yes	16	3	89%	Low
Morandeira-Rivas et al., 2012 [75]	Yes	Yes	No	Yes	Yes	Yes	No	Yes	Yes	Yes	Yes	Yes	No	NA	Yes	Yes	Yes	Yes	No	Yes	16	3	89%	Low
O’Sullivan 2002 [62]	Yes	Yes	No	Yes	Yes	Yes	No	Yes	Yes	No	Yes	Yes	No	NA	Yes	Yes	Yes	Yes	No	Yes	15	4	84%	Low
Plerhoples et al., 2012 [20]	Yes	Yes	No	Yes	Yes	Yes	No	Yes	Yes	Yes	Yes	Yes	No	NA	Yes	Yes	Yes	Yes	No	Yes	16	3	89%	Low
Quinn and Moohan 2015 [72]	Yes	Yes	No	Yes	Yes	Yes	No	Yes	Yes	Yes	Yes	Yes	No	NA	Yes	Yes	Yes	No	No	Yes	15	4	84%	Low
Rambabu and Suneetha 2014 [23]	Yes	Yes	No	Yes	Yes	Yes	No	Yes	Yes	No	Yes	Yes	No	NA	Yes	Yes	Yes	Yes	No	Yes	15	4	84%	Low
Rata et al., 2021 [25]	Yes	Yes	No	Yes	Yes	Yes	No	Yes	Yes	Yes	Yes	Yes	No	NA	Yes	Yes	Yes	Yes	No	Yes	16	3	89%	Low
Riaz et al., 2021 [83]	Yes	Yes	Yes	Yes	Yes	Yes	No	Yes	Yes	Yes	Yes	Yes	No	NA	Yes	Yes	Yes	Yes	No	Yes	17	2	95%	Low
Ruitenburg et al., 2012 [76]	Yes	Yes	No	Yes	Yes	Yes	No	Yes	Yes	Yes	Yes	Yes	No	NA	Yes	Yes	Yes	No	No	Yes	15	4	84%	Low
Sari et al., 2010 [77]	Yes	Yes	No	Yes	Yes	Yes	No	Yes	Yes	Yes	Yes	Yes	No	NA	Yes	Yes	Yes	Yes	No	Yes	16	3	89%	Low
Sivak-Callcott et al., 2011 [58]	Yes	Yes	No	Yes	Yes	Yes	No	Yes	Yes	Yes	Yes	Yes	No	NA	Yes	Yes	Yes	No	No	Yes	15	4	84%	Low
Stomberg et al., 2010 [21]	Yes	Yes	No	Yes	Yes	Yes	No	Yes	Yes	Yes	Yes	Yes	No	NA	Yes	Yes	Yes	Yes	No	Yes	16	3	89%	Low
Szeto et al., 2009 [64]	Yes	Yes	No	Yes	Yes	Yes	No	Yes	Yes	Yes	Yes	Yes	No	NA	Yes	Yes	Yes	Yes	No	Yes	16	3	89%	Low
Tan and Kwek 2020 [26]	Yes	Yes	No	Yes	Yes	Yes	No	Yes	Yes	Yes	Yes	Yes	No	NA	Yes	Yes	Yes	Yes	No	Yes	16	3	89%	Low
Tjiam et al., 2014 [78]	Yes	Yes	No	Yes	Yes	Yes	No	Yes	Yes	Yes	Yes	Yes	No	NA	Yes	Yes	Yes	Yes	No	Yes	16	3	89%	Low
Trejo et al., 2006 [59]	Yes	Yes	No	Yes	Yes	Yes	No	Yes	Yes	Yes	Yes	Yes	No	NA	Yes	Yes	Yes	No	No	Yes	15	4	84%	Low
Vaghela et al., 2019 [24]	Yes	Yes	No	Yes	Yes	Yes	No	Yes	Yes	No	Yes	Yes	No	NA	Yes	Yes	Yes	Yes	No	Yes	15	4	84%	Low
Vijendren et al., 2016 [81]	Yes	Yes	No	Yes	Yes	Yes	No	Yes	Yes	No	Yes	Yes	No	NA	Yes	Yes	Yes	Yes	No	Yes	15	4	84%	Low
Voss et al., 2016 [3]	Yes	Yes	No	Yes	Yes	Yes	No	Yes	Yes	Yes	Yes	Yes	No	NA	Yes	Yes	Yes	Yes	No	Yes	16	3	89%	Low
Wohlauer et al., 2021 [36]	Yes	Yes	No	Yes	Yes	Yes	No	Yes	Yes	Yes	Yes	Yes	No	NA	Yes	Yes	Yes	Yes	No	Yes	16	3	89%	Low
Wolf et al., 2000 [13]	Yes	Yes	No	Yes	Yes	Yes	No	Yes	Yes	Yes	Yes	Yes	No	NA	Yes	Yes	Yes	No	No	Yes	15	4	84%	Low

*: An absence of conflict (No answer) is considered a mark of quality and is therefore counted as a Yes in the computation of the quality score.

**Table 4:** Continental rankings by WMSD prevalence, overall and by body area.

Rank	1	2	3
**Neck**	Europe (48.8%)	Asia (41.5%)	America (40.2%)
**Upper back**	Europe (36.5%)	Asia (29.0%)	America (28.9%)
**Lower back**	Asia (45.4%)	Europe (39.6%)	America (38.2%)
**Shoulder**	Europe (42.7%)	America (30.3%)	Asia (28.1%)
**Elbow**	America (13.9%)	Europe (12.6%)	Asia (9.0%)
**Wrist**	Europe (26.7%)	America (25.2%)	Asia (21.4%)
**Hip**	Asia (12.1%)	America (7.7%)	-
**Knee**	Europe (23.8%)	Asia (17.8%)	America (12.8%)
**Ankle**	Asia (20.1%)	Europe (13.5%)	America (12.4%)
**Overall**	Asia (77.6%)	Europe (73.1%)	America (62.8%)

**Table 5:** Ranking of WMSD prevalence by body area for each continent.

Rank	America	Asia	Europe
**1**	Neck (40.2%)	Lower back (45.4%)	Neck (48.8%)
**2**	Lower back (38.2%)	Neck (41.5%)	Shoulder (42.7%)
**3**	Shoulder (30.3%)	Upper back (29.0%)	Lower back (39.6%)
**4**	Upper back (28.9%)	Shoulder (28.1%)	Upper back (36.5%)
**5**	Wrist (25.2%)	Wrist (21.4%)	Wrist (26.7%)
**6**	Elbow (13.9%)	Ankle (20.1%)	Knee (23.8%)
**7**	Knee (12.8%)	Knee (17.8%)	Ankle (13.5%)
**8**	Ankle (12.4%)	Hip (12.1%)	Elbow (12.6%)
**9**	Hip (7.7%)	Elbow (9.0%)	-

## Data Availability

Data available on request.

## Abbreviations

The following abbreviations are used in this manuscript:

WMSDs	Work-related musculoskeletal disorders
AXIS	Tool for evaluating cross-sectional studies
BMI	Body mass index
PRISMA	Preferred Reporting Items for Systematic reviews and Meta-Analyses
